# FunctionAnnotator, a versatile and efficient web tool for non-model organism annotation

**DOI:** 10.1038/s41598-017-10952-4

**Published:** 2017-09-05

**Authors:** Ting-Wen Chen, Ruei-Chi Gan, Yi-Kai Fang, Kun-Yi Chien, Wei-Chao Liao, Chia-Chun Chen, Timothy H. Wu, Ian Yi-Feng Chang, Chi Yang, Po-Jung Huang, Yuan-Ming Yeh, Cheng-Hsun Chiu, Tzu-Wen Huang, Petrus Tang

**Affiliations:** 1 Bioinformatics Center, Chang Gung University, Taoyuan, Taiwan; 2 Molecular Medicine Research Center, Chang Gung University, Taoyuan, Taiwan; 3 Graduate Institute of Biomedical Sciences, College of Medicine, Chang Gung University, Taoyuan, Taiwan; 4 Department of Biochemistry and Molecular Biology, College of Medicine, Chang Gung University, Taoyuan, Taiwan; 5 Department of Otolaryngology - Head & Neck Surgery, Chang Gung Memorial Hospital, Taoyuan, Taiwan; 6 Institute of Biomedical Informatics, National Yang-Ming University, Chang Gung Memorial Hospital, Taoyuan, Taiwan; 7 Molecular Infectious Diseases Research Center, Chang Gung Memorial Hospital, Taoyuan, Taiwan; 8 Department of Microbiology and Immunology, School of Medicine, College of Medicine, Taipei Medical University, Taipei, Taiwan; 9 Molecular Regulation & Bioinformatics Laboratory, Chang Gung University, Taoyuan, Taiwan; 10 Clinical Proteomics Core Laboratory, Chang Gung Memorial Hospital, Taoyuan, Taiwan; 11 Center for General Education, Chang Gung University, Taoyuan, Taiwan; 12 Department of Colorectal Surgery, Chang Gung Memorial Hospital, Taoyuan, Taiwan

## Abstract

Along with the constant improvement in high-throughput sequencing technology, an increasing number of transcriptome sequencing projects are carried out in organisms without decoded genome information and even on environmental biological samples. To study the biological functions of novel transcripts, the very first task is to identify their potential functions. We present a web-based annotation tool, FunctionAnnotator, which offers comprehensive annotations, including GO term assignment, enzyme annotation, domain/motif identification and predictions for subcellular localization. To accelerate the annotation process, we have optimized the computation processes and used parallel computing for all annotation steps. Moreover, FunctionAnnotator is designed to be versatile, and it generates a variety of useful outputs for facilitating other analyses. Here, we demonstrate how FunctionAnnotator can be helpful in annotating non-model organisms. We further illustrate that FunctionAnnotator can estimate the taxonomic composition of environmental samples and assist in the identification of novel proteins by combining RNA-Seq data with proteomics technology. In summary, FunctionAnnotator can efficiently annotate transcriptomes and greatly benefits studies focusing on non-model organisms or metatranscriptomes. FunctionAnnotator, a comprehensive annotation web-service tool, is freely available online at: http://fa.cgu.edu.tw/. This new web-based annotator will shed light on field studies involving organisms without a reference genome.

## Introduction

With the improvement of sequencing technology, Next-Generation Sequencing (NGS) has been used increasingly frequently for transcriptome studies^1^. Analyzing transcriptomes from non-model organisms is very different from that of model organisms because of the lack of proper reference genomes. Several methods have been proposed to assemble transcripts from sequencing reads without a reference genome, such as Trinity, Oases and SOAPdenovo^2–4^, etc. The next step following transcript assembly is to annotate functions of these transcripts, and many tools are proposed for this purpose. For example, RAST (Rapid Annotation using Subsystem Technology) provides annotations for bacterial and archaeal genomes^5, 6^. Blast2GO annotates Gene Ontology (GO) terms^7^ based on BLAST search results^8^. Other tools, such as TMHMM for transmembrane protein prediction^9^, SignalP for signal peptide prediction^10^, LipoP for lipoprotein identification^11^, and PSORT for subcellular localization characterization^12, 13^, utilize sequence features for functional annotation. These tools have already been available for a long time. However, many of these tools demand significant computing skills from users, and familiarity with a command line environment is often a necessity. Hence a user-friendly annotation tool will be beneficial for all of these transcriptome studies.

In 2012, we published the web server FastAnnotator^14^, which aims to annotate transcript contigs assembled from RNA-Seq reads. It has been widely used and has provided annotation for more than 1,500 projects. Recently, TRUFA, an RNA-Seq analysis tool specifically designed for non-model organisms has been proposed^15^. While TRUFA involves the entire RNA-Seq analysis process, there is less emphasis on annotation. We believe that it is very important to offer annotations for potential functions for those transcriptomes lacking reference genomes. Therefore, we propose a successor to FastAnnotator, FunctionAnnotator, which focuses on providing comprehensive functional annotations and generating more output files that could be valuable in further downstream analyses. FunctionAnnotator includes annotations for GO terms, enzyme identification, domain detection, lipoprotein recognition, transmembrane domain discovery, subcellular localization annotation, etc. FunctionAnnotator also provides the distribution of species from best hits at different taxonomic levels. All of these annotation results can be downloaded as a text file for further analyses or integrated with experiments other than sequencing.

Another emerging field requiring annotation for transcriptomes is metatranscriptome analysis^16–19^. Functional annotation of metatranscriptomes can reveal which pathways and genes are highly expressed in the environmental sample at a specific time and place^20^. In addition to functional annotations, Leimena *et al*. have demonstrated that there is a high agreement between community composition profiles derived from 16 S rRNA qPCR and metatranscriptomic data^21^. Therefore, metatranscriptomics can also be a surrogate for metagenomics, in terms of its potential for understanding the community composition of environmental samples. Some studies propose methods for analyzing these metatranscriptomic data^19, 22, 23^ and analysis pipeline such as SAMSA was proposed. One feasible approach is to search for homologs in the NCBI NR database using all of the transcripts. By identifying the species with the most similar hits and obtaining taxonomic information for these species, users can have a phylogenetic profile similar to that derived from metagenomics analysis and have a global idea about the potential composition of species in the original community. Therefore, we also implemented this strategy to generate an estimation of the distribution of species in the original samples, based on a homology search in FunctionAnnotator. Our design enables FunctionAnnotator to disclose species distribution, functions for transcripts and all of the activated pathways hidden in the metatranscriptomic data.

In this study, we present the web tool FunctionAnnotator and prove that FunctionAnnotator can annotate and provide community composition for metatranscriptomics. In another example, we further showed that the output from FunctionAnnotator can assist other relative experiments such as proteomics analysis. In summary, FunctionAnnotator guarantees an easy-to-use method for understanding the transcriptomes of non-model organisms and produce annotations and predictions, which may open many possibilities for further application or integration with other fields of study. We herein have developed a trouble-free solution for the analysis of transcriptomes from non-model organisms.

## Results and Discussion

### FunctionAnnotator provides comprehensive and efficient annotation for transcriptomes from non-model organisms

**Figure 1:**
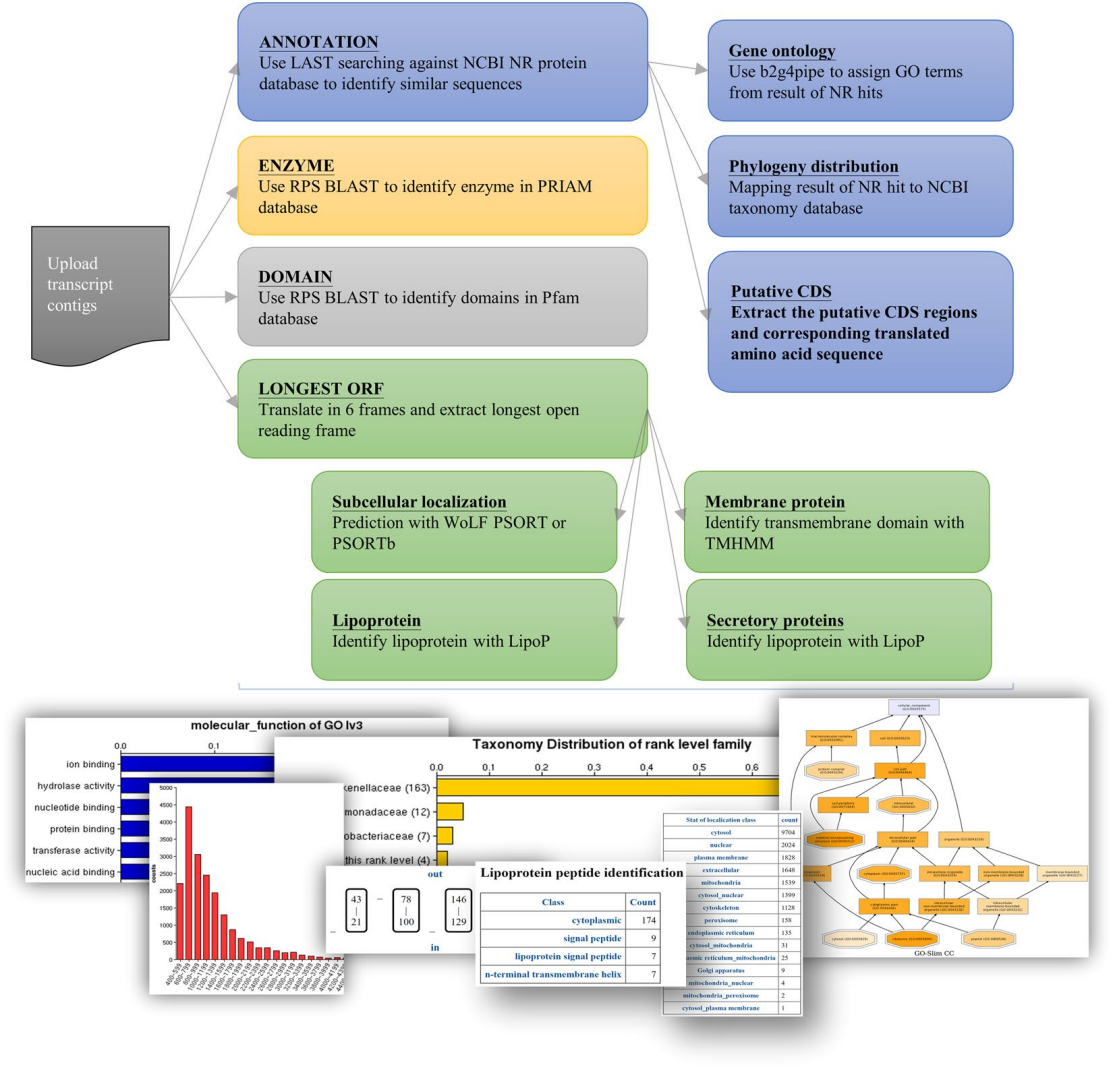
Annotation system implemented in FunctionAnnotator. After users upload a FASTA file containing nucleotide sequences and select the desired analysis modules, FunctionAnnotator will execute all of the selected annotation processes in parallel. FunctionAnnotator includes in-house scripts and annotation tools, as listed in this figure, including LAST, BLAST2GO, PSORT, TMHMM, etc. for annotating GO terms, enzyme and domain identification, predictions for subcellular localization, lipoproteins, secretory proteins and transmembrane proteins, etc. For each annotation category, FunctionAnnotator annotates uploaded sequences with corresponding annotation tools and integrates the output into graphs or tables. All of the annotation results are also available for download as text files.

**Figure 2:**
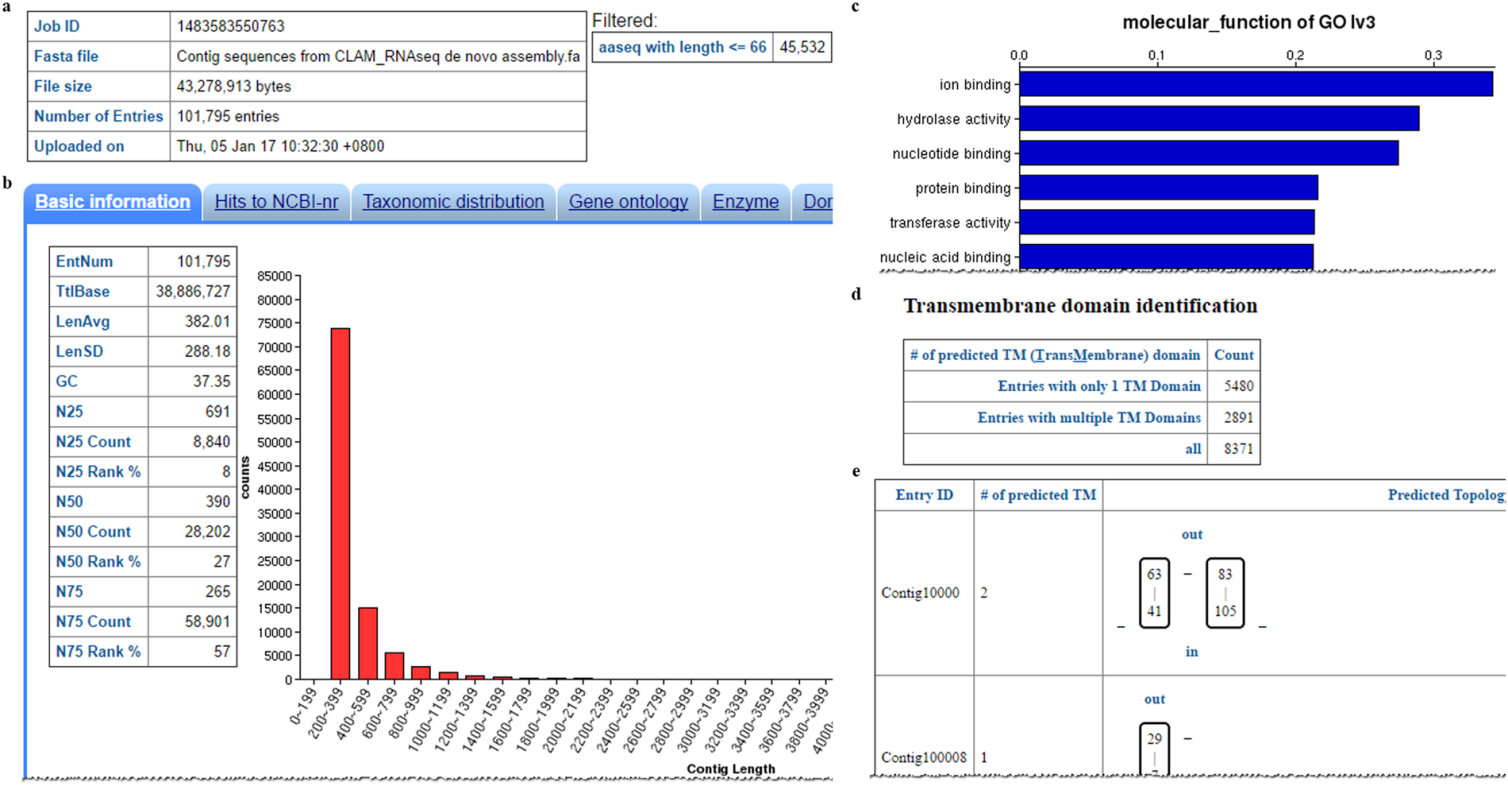
Partial annotation result for the clam transcriptome. (**a**) Basic statistics for uploaded nucleotide sequences including number of entries (contigs), total base pairs and upload date are listed in the table. (**b**) Basic information from the uploaded contigs, including GC content, N50, average length, etc., are listed in this table together with a bar chart of the length distribution for contigs. (**c**) Distribution of GO annotation results for molecular function. The most abundant molecular function in the 3^rd^ level is ion binding, which can be found in approximately 34% of GO annotated contigs. Of note, each contig can have more than one GO term assignment, therefore the total percentage from this bar chart is larger than 1. (**d**) Transmembrane domain (TM) prediction results show 5,480 contigs have one TM domain and 2,891 contigs have multiple TM domains. FunctionAnnotator also plots the predicted topology of transmembrane domains along with their positional information.

The overall annotation system built into FunctionAnnotator is illustrated in Fig. 1. To examine the performance and efficiency of FunctionAnnotator, four assembled transcriptomes from different non-model organism datasets ranging from 38 Mb to 0.85 Mb were used as examples (Table 1). FunctionAnnotator finished all annotations, including GO term assignment, enzyme annotation, domain identification, predictions for subcellular localization, lipoprotein, secretory protein and transmembrane protein, etc., with 7 and half hours for transcripts with a total length of 38 Mb from clams (*Meretrix meretrix*). Parallel computing in FunctionAnnotator sped the annotation processes and cut down the computing time to less than half of the time that FastAnnotator^14^ required. Furthermore, with the most updated database and integration of more functional prediction tools (including taxonomic distribution, transmembrane domain, subcellular localization, lipoprotein and signal peptide prediction), FunctionAnnotator provides functional annotation for 35,971 contigs out of 56,263 contigs that have predicted amino acid sequences of more than 66 amino acids. Only the 35,971 contigs are annotated because there are only few annotated genes encode less than 67 amino acids^24^ and contigs can’t produce a product longer than 66 amino acids are likely derived from insufficient number of reads. FunctionAnnotator also provides potential subcellular localizations for the encoded proteins from all these 56,263 contigs. All the basic statistics for uploaded contigs and features of contigs are also presented in the tables and figures as shown in Fig. 2a,b.

**Table 1:** Benchmarks for FunctionAnnotator performance.

Organism(s)	# of contigs	Total bp	# of contigs with best hit (%)	# of contigs annotated^*^ (%)	Elapsed Time
Clam	101,795	38,886,727	29,960 (29%)	35,971 (64%)	7 h 20 m 38 s
Metatranscriptome I	241	85,193	225 (93%)	126 (64%)	24 m 47 s
Metatranscriptome II	381	137,588	367 (96%)	243 (76%)	29 m 57 s
Trichomonas	19,415	24,204,403	16,866 (87%)	13,497 (70%)	3 h 26 m 56 s

^*^Only contigs having predicted coding sequences longer than 66 were counted and subcellular localization prediction results are eliminated.

**Figure 3:**
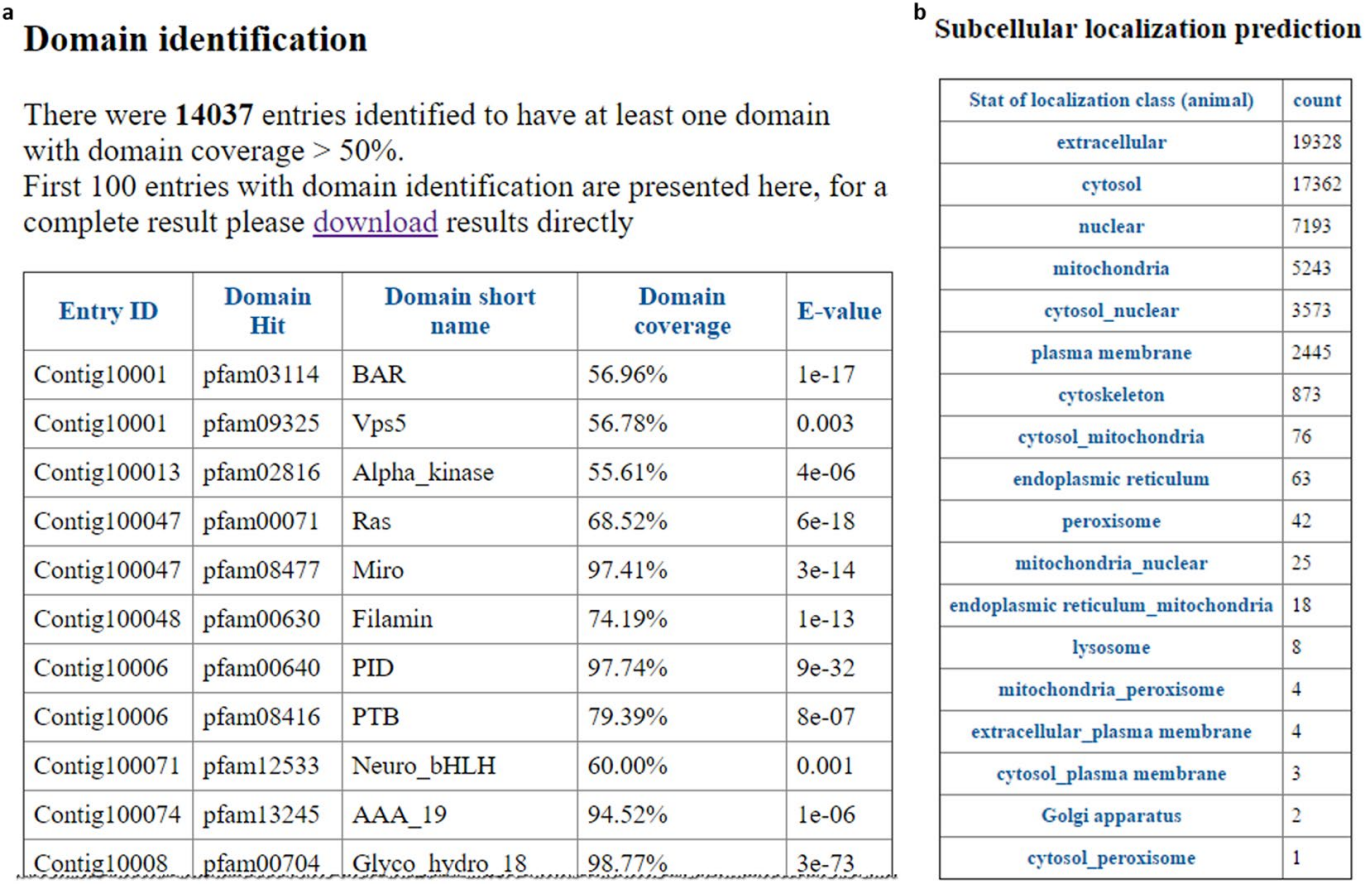
Domains and subcellular localization predictions for transcripts from clam. (**a**) Domain identification result (partial) shows that FunctionAnnotator identified 14,037 domains from this transcriptome. The identified domains are shown together with their domain IDs, domain names, domain coverages and RPS BLAST e-values. (**b**) Subcellular localization prediction results demonstrate that 19,339 of the transcripts are predicted to be located in the extracellular compartment followed by 17,362 transcripts located in the cytosol. FunctionAnnotator presents this summary table and a detailed table containing subcellular localization and a prediction score for each contig.

From the functional annotation, the clam transcriptome was found to be enriched in contigs that have a “binding” molecular function. From GO term annotation, we found that the most abundant molecular functions in this clam transcriptome are ion binding, hydrolase activity, nucleotide binding, protein binding, transferase activity and nucleic acid binding (Fig. 2c). These results are consistent with previous studies, which show that the most abundant molecular function for transcripts is “binding” in clam (*Meretrix meretrix*), whelk (*Rapana venosa*), Eastern oyster (*Crassostrea virginica*) and Pacific oyster (*Crassostrea gigas*)^25–28^. Of note, using the same analysis strategy as FastAnnotator^14^, FunctionAnnotator provides GO term annotations and allows users to select the level of GO term they want to explore. Users can select any level, and the new distribution will be shown in the bar chart instantaneously. In the clam annotation results, if one selects level 2 on the output page, the most dominant molecular function will change to “binding”. Moreover, cation channels are proposed to be involved in the response to osmotic stress for these marine creatures^25^, and indeed, we found almost one quarter (8,371 out of 35,971) of the annotated contigs contain at least one transmembrane domain (Fig. 2d). FunctionAnnotator also illustrates the predicted topology for these predicted transmembrane proteins (Fig. 2e). In addition to transmembrane domains, FunctionAnnotator also identifies domains in transcripts. In this transcriptome, FunctionAnnotator identified domains from 14,037 entries (Fig. 3a), among which 2,299 entries do not have similar sequences in the NR database. These 2,299 entries may be incomplete transcripts derived from low coverage transcripts in *de novo* assembly or novel genes that have conserved domains combined with other new sequences. This domain identification strategy can therefore increase the likelihood of identifying potential functions. As for subcellular localization prediction, FunctionAnnotator reports the predicted localizations with the highest scores for contigs and presents the results together with prediction scores in the table on the output page. For eukaryote samples, FunctionAnnotator shows prediction for animal, plant and fungi and user can choose the most fitting category by themselves (Fig. 3b).

### FunctionAnnotator is also beneficial for understanding metatranscriptomes

**Figure 4:**
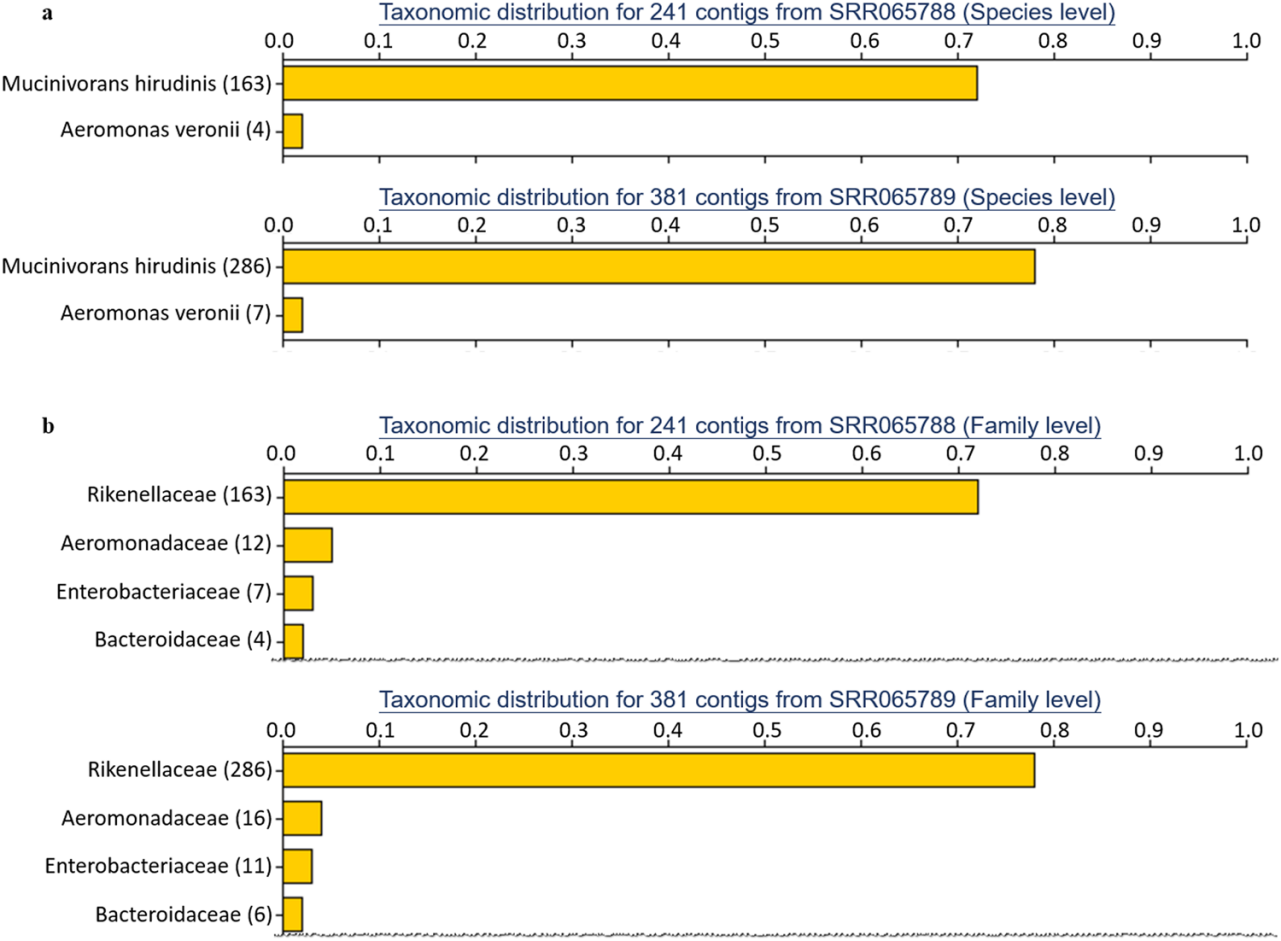
Taxonomy distribution for two metatranscriptomes from the gut microbiome of medicinal leech. FunctionAnnotator searched the NR database for a homolog of each transcript and then identifies which species the best hits come from. The taxonomic information for these species is presented in a bar chart and the user can select different taxonomic levels. (**a**) At the species level, the best hits of 163 out of the original 241 contigs are from *Mucinivorans hirudinis*, and for another 4 contigs, the best hits are from *Aeromonas veronii* for the first dataset. Similar results were obtained for the second dataset. (**b**) At the Family level, again the most abundant family is *Rikenellaceae*, followed by *Aeromonadaceae*, *Enterobacteriaceae* and *Bacteriaoidaceae* for both metatranscriptomes.

We also implemented taxonomic classification in FunctionAnnotator and explored the potential of FunctionAnnotator in analysis of metatranscriptomes. FunctionAnnotator identifies which species the best hits come from and uses a pre-calculated taxonomy tree to provide taxonomy information at different levels including species, genus, family, order, class, phylum and kingdom. A similar visualization strategy used for GO distribution is implemented for displaying the taxonomic distribution, which will change accordingly when users select a different taxonomic level. Here, we used two metatranscriptome datasets from a previous study by Bomar *et al*.^16^ to test how helpful FunctionAnnotator can be in analyzing taxonomic distribution. We used the same tools as Bomar *et al*. (CLC Genomics Workbench) to assemble contigs from RNA-Seq reads downloaded from GSE23786 in the NCBI GEO database^29, 30^. There were two samples in GSE23786, SRR065788 and SRR065789. Both samples are metatranscriptomes of gut microbiomes from the medicinal leech *Hirudo verbana* and are listed as Metatranscriptome I and Metatranscriptome II, respectively, in Table 1. FunctionAnnotator successfully identified the most abundant species as *Mucinivorans hirudinis* and the second most abundant species as *Aeromonas veronii*, in both datasets (Fig. 4a). Previously, Nelson *et al*. had identified *Mucinivorans hirudinis* as a new genus within the *Rikenellaceae* ^31^ family. We also found that at the family level, the most abundant family is *Rikenellaceae* in FunctionAnnotator (Fig. 4b). In Bomar’s report, they also claim that the most abundant species is uncultured *Rikenella*-like bacterium followed by *A. veronii* ^16^. FunctionAnnotator generates the same conclusion with even more precise taxonomic distribution because of the updated NR database. Hence, we demonstrated that FastAnnotator provides a practical solution for identifying community composition in metatranscriptomes. This result is encouraging and suggests that our strategy can potentially identify relatives of transcripts from uncultured bacteria. Even though these uncultured bacteria may have few or even no sequence records in the NR database, FunctionAnnotator can utilize homologs from other bacterial species belonging to the same family.

**Figure 5:**
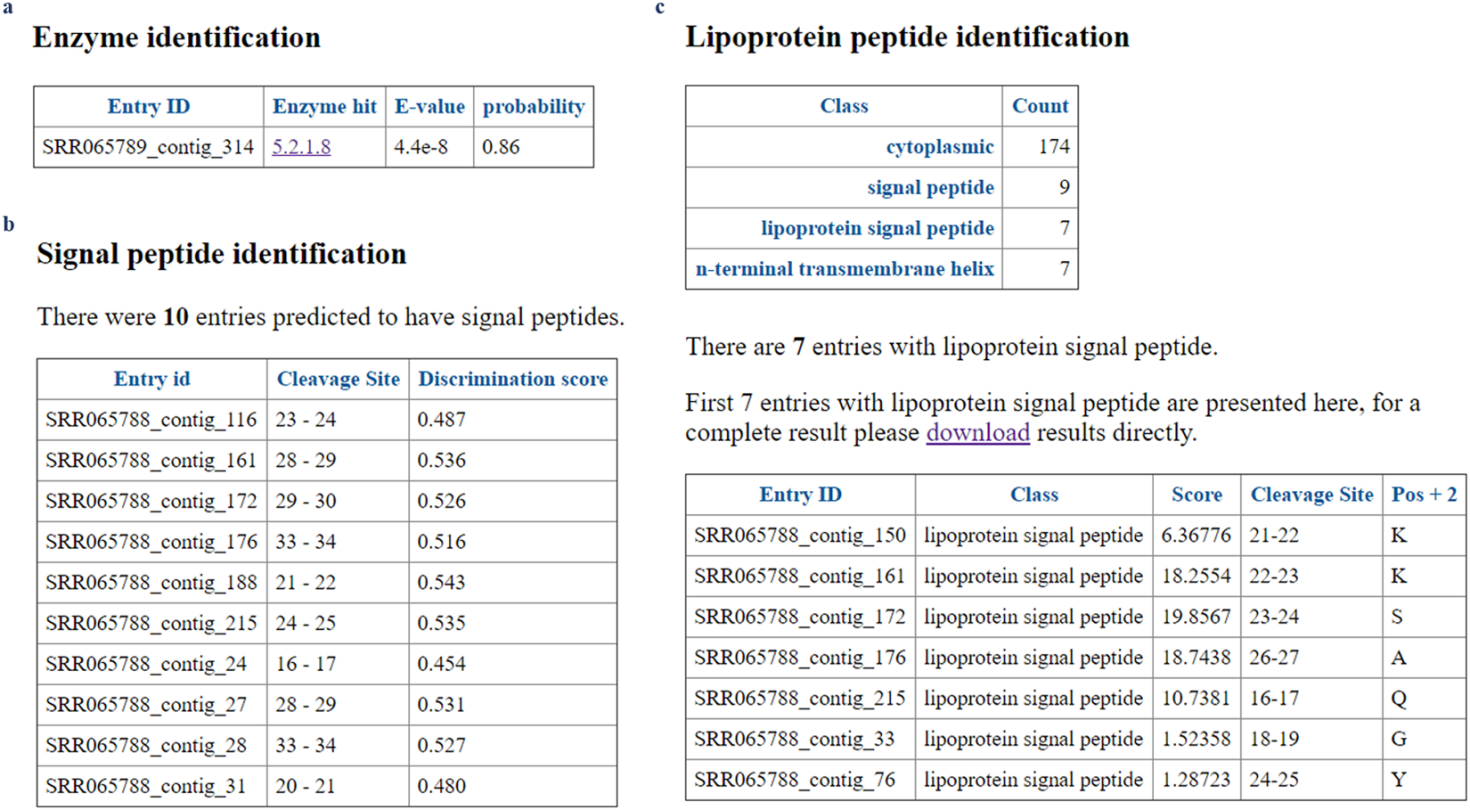
Enzyme, lipoprotein and signal peptide identification for metatranscriptomes from the gut microbiome of medicinal leech. (**a**) One putative enzyme identified in this metatranscriptome listed together with its predicted EC number. By clicking on the EC number, the user will be linked to a website providing more detailed information about the chemical reactions the enzyme catalyzes. (**b**) Putative signal peptides identified by FunctionAnnotator are also listed, as well as their predicted cleavage sites and prediction scores. (**c**) Putative lipoproteins are listed with predicted score, cleavage site and the amino acid in position +2 after the cleavage site.

In addition to the community composition, FunctionAnnotator also annotated these two metatranscriptomes and found potential functions for 64% and 76% of contigs, respectively (Table 1). The annotation result also identified many hydrolytic enzymes and transporters, which are proposed to provide clues for modifying culture medium in order to isolate these *Rikenella*-like bacteria^16^. One enzyme was identified in the SRR065789 dataset (Fig. 5a). FunctionAnnotator also provides links to the ExPASy database^32^, providing detailed descriptions about enzyme activity and may thus offer more detailed information about the metabolic activity within these *Rikenella*-like bacteria. Another annotation offered in FunctionAnnotator is the identification of signal peptides (Fig. 5b), which predict potential secretory or transmembrane proteins. Moreover, FunctionAnnotator also identified lipoproteins from bacterial transcripts (Fig. 5c) with LipoP, which claimed to identify lipoproteins with a sensitivity as high as 96.8% and a false positive rate as low as 0.3%^11^. Seven and eleven lipoproteins were identified in these two metatranscriptome datasets. The identification of lipoproteins can be meaningful in pathogenic bacteria, as many lipoproteins are known to play an important role in virulence and are involved in host-pathogen interactions^33^. Taken together, all of these results support FunctionAnnotator being a useful tool for metatranscriptome analysis.

We further compared the performance of FunctionAnnotator with previous metatranscriptome works. We carried out similar analysis with the four transcriptomes provided by Leimena *et al*. (SRP020487)^21^. From the assembled contigs, FunctionAnnotator identified the same top five dominant genus with fewer unclassified genus, which may again due to the updated database (Supplementary Figure 1a). We also analyzed the same dataset by using SAMSA, which utilize MG-RAST^34^ for annotating. Our results demonstrated that the organism distribution profile is similar to that identified by FunctionAnnotator (Supplementary Figure 1b).

### Performance of FunctionAnnotator on simulated datasets

In addition to comparing with previous metatranscriptome analysis results and tools, we also tested the performance of FunctionAnnotator on simulated datasets. Three simulated transcriptomes from *Sulfolobus tokodaii*, *Streptomyces coelicolor* and *Yersinia pestis* generated by Grinder^34^ were annotated by FunctionAnnotator. FunctionAnnotator identified correct taxonomy for almost all of the contigs at the genus level, but not the species level (Supplementary Figure 2–4) due to some contigs have best hits in other closely related organisms in the NR database. These results demonstrated that FunctionAnnotator can provide correct taxonomic assignment for almost all of the transcriptome from a single organism. We also tested FunctionAnnotator with 12 simulated metatranscriptomes, from 5, 10, 20 and 50 randomly selected organisms (Supplementary Table 1–4). FunctionAnnotator identified all genus from these simulated metatranscriptome datasets (Supplementary Figure 5, 6). We conclude that FunctionAnnotator can assign contigs with the correct taxonomy groups at the genus level for metatranscriptomes.

### Annotation results from FunctionAnnotator can benefit proteomics analysis

While an increasing number transcriptome sequencing projects are proposed and carried out as sequencing technology improves, some of them are also accompanied by proteomics analysis. While annotating transcripts, FunctionAnnotator also generates putative amino acid sequences based on homology searches in the NR database. These sequences could be helpful in downstream follow up analysis such as protein identification. We used an example from *Trichomonas tenax* to demonstrate how FunctionAnnotator can be useful in analyzing proteomics data. *T. tenax* is an anaerobic protist commonly found in the human oral cavity and possesses a mitochondria-related organelle, termed the hydrogenosome, instead of a mitochondrion^35^. Previous studies in *T. vaginalis* showed that the functions of a hydrogenosome include ATP production, iron-sulfur cluster assembly, anti-oxidative stress and some amino acid metabolism^36^. As it lacks a complete genome, *T. tenax* is a perfect example dataset for utilizing FunctionAnnotator to annotate its^37^ *tenax* with FunctionAnnotator. Later, we used nucleotide sequences from contigs or predicted amino acid sequences from FunctionAnnotator as its surrogate proteome reference database.

From the proteomics data, we were able to identify 1,434 proteins by LC/MS with the amino acid RNA-Seq dataset as the reference database^37^. Proteins involved in ATP production, iron-sulfur cluster assembly, as well as other known hydrogenosomal functions, were the best hits identified in our proteome results. For instance, 14 proteins have been shown to be involved in *T. vaginalis* iron-sulfur cluster assembly to date and we identified 11 of them (IscA, IscS, frataxin, ferredoxin, HydE, HydF, HydG, HSP70, Jac1, Mge, and Ind). Only Nfu, IscU, and Isd11 were missing in our proteome data. Additionally, all ATP production-related enzymes except succinyl-CoA synthetase α subunit (SCSα) were identified. It is worth mentioning that when using predicted amino acid sequences as a search database, we can identify approximately 10% more peptides than using only contig sequences. This increase in sensitivity results from a smaller number of sequences in the surrogate reference database. Hence, we have shown that the predicted amino acid sequences produced by FunctionAnnotator based on homology searches can improve the sensitivity of protein identification in analyzing LC/MS data.

## Materials and Methods

### Identification of GO terms, domains, enzymes and best protein hits in the NR database

FunctionAnnotator provides GO term assignment and domain and enzyme identification by employing the same strategies as FastAnnotator^8, 14, 38–40^. In short, we implement some mathematical transformations to accelerate the annotation process. For all of the above analysis, FunctionAnnotator uses updated databases for GO terms^41^, Pfam^42^, PRIAM^43^ and the NR database^44^. Putative CDS and the corresponding translated amino acid sequences were further extracted and translated *in silico* from LAST homology search results^39, 40^. These sequences are presented in FASTA format and are included in the zipped file for download.

### Taxonomic analysis for organisms with the best contigs hits in the NR database

LAST^39, 40^, which was shown comparable and faster than BLASTX^14^, was used to identify the most similar sequences in the NCBI NR database^44^ for each contig. In house scripts were used to identify which species the best hit sequence come from and the taxonomic information for that particular species. We also implemented a built-in pre-computed taxonomy tree structure in our database for re-calculating species distribution at different taxonomic levels.

### Identification of membrane proteins, lipoproteins and secretory proteins

FunctionAnnotator utilizes TMHMM 2.0c^9^, SignalP 4.1^10^ and LipoP 1.0a^11^, to identify transmembrane proteins, signal peptides and lipoproteins, respectively. Specifically, FunctionAnnotator applies six-frame translation and uses the longest open reading frame (ORF) for all uploaded contigs for potential transmembrane domain, lipoprotein or single peptide prediction. Of note, contigs that have the longest predicted ORF shorter than 198 bp (66 amino acid) are filtered out. Membrane protein predictions are available for samples from all three kingdoms (bacteria, archaea and eukaryote) with TMHMM which has the high sensitivity and specific and is the most commonly used transmembrane protein prediction tool^9, 45, 46^. It is worth mentioning that there are several lipoprotein prediction tools proposed, including LipoP, PRED-LIPO and LipPred^11, 47, 48^. However, only LipoP provides source code and it is the most widely used lipoprotein prediction tool. Additionally, even though LipoP is originally designed for lipoprotein prediction in Gram-negative bacteria, it has been demonstrated to perform well for prediction of lipoproteins in Gram-positive bacteria, as well^11, 49^. Therefore, FunctionAnnotator uses LipoP to predict lipoproteins for all bacteria samples. For signal peptide prediction, one of the most commonly used and accurate signal peptide prediction tool, SignalP 4.1^10^ together with appropriate organism group (Eukaryotes, Gram-positive bacteria or Gram-negative bacteria) parameter is used to identify potential secretory proteins.

### Prediction of subcellular localization

FunctionAnnotator exploits WoLF PSORT 0.2 and PSORTb 3.0 for prediction of subcellular localization for eukaryotes and bacteria, respectively^12, 13^. Both PSORTb and WoLF PSORT trained their algorithms with SWISS-Prot and show high precision and recall^12, 13, 50^. These two tools are also the most widely used subcellular localization prediction tools. PSORT predicts subcellular localization by searching for signals, amino acid composition and motifs from the amino acid sequences of the predicted protein product from contigs. Potential subcellular localizations include chloroplast, cytosol, cytoskeleton, endoplasmic reticulum, extracellular, Golgi apparatus, lysosome, mitochondria, nuclear, peroxisome, plasma membrane and vacuolar membrane. Only the predicted location with the highest score for each contig is listed in the output table. All the prediction scores together with the predicted subcellular localizations are parsed and presented in a summary table.

### Implementation

To provide an efficient web-server, all the processes used for analysis have been paralleled, and the server handles two projects at once. Other submitted jobs are listed in a first-come, first-served queuing system. After the FASTA file is uploaded, FunctionAnnotator checks whether these sequences were fully composed of nucleotide sequences and eliminates contigs containing any bases other than “A”, “T”, “C”, “G” or “N”. Several in house scripts written in Perl or Python are used to integrate all of the annotation results. The FunctionAnnotator website was constructed with PHP and JavaScript.

### Simulation of transcriptome and metatranscriptome data

Transcriptomes of 2,774 completely sequenced and annotated bacteria genomes were downloaded from the NCBI genomes ftp site (https://ftp.ncbi.nih.gov/genomes/). We randomly selected three organisms, *Sulfolobus tokodaii*, *Streptomyces coelicolor* and *Yersinia pestis* for transcriptome simulation. Grinder^35^ was used to generate 0.02 million reads for each organism. We also created 12 metatranscriptome datasets by combining 5, 10, 20 and 50 randomly selected bacteria transcriptomes as shown in Supplementary Table 1–4. Each metatranscriptome dataset contain 1 million simulated reads. For all simulated datasets, the length of reads were 300 bp with default Phred quality scores range.

### Availability

FunctionAnnotator is freely available at http://fa.cgu.edu.tw. The website can be accessed by popular web browsers with JavaScript enabled, including Mozilla Firefox, Google Chrome and Microsoft Internet Explorer.

## Electronic supplementary material

Supplementary Fig. 1-6 and Supplementary Table 1-4
