# Supplementary material for: FunctionAnnotator, a versatile and efficient web tool for non-model organism annotation
Source: Sci Rep. 2017 Sep 5;7:10430. doi: 10.1038/s41598-017-10952-4 (PMC5585236; doi:10.1038/s41598-017-10952-4)
Supplement: Supplementary file 1 — Supplementary Fig. 1-6 and Supplementary Table 1-4 [file 41598_2017_10952_MOESM1_ESM.pdf]

FunctionAnnotator, a versatile and efficient web tool for non-model organism annotation

Ting-Wen Chen[1,2*], Ruei-Chi Gan[1,2*], Yi-Kai Fang[3], Kun-Yi Chien[4], Wei-Chao Liao[2,5], Chia-Chun Chen[4], Timothy H. Wu[6], Ian Yi-Feng Chang[1,2], Chi Yang[1,2], Po-Jung Huang[1,2], Yuan-Ming Yeh[1,2], Cheng-Hsun Chiu[7], Tzu-Wen Huang[8] and Petrus Tang[1,7,9§]

[1]Bioinformatics Center, [2]Molecular Medicine Research Center, [3]Graduate Institute of Biomedical Sciences, College of Medicine, [4]Proteomics Core Laboratory, and [9]Molecular Regulation & Bioinformatics Laboratory, Chang Gung University, Taoyuan, Taiwan. [6]Institute of Biomedical Informatics, National Yang-Ming University, [5]Department of Otolaryngology - Head & Neck Surgery, [7]Molecular Infectious Diseases Research Center, Chang Gung Memorial Hospital, Taoyuan, Taiwan. [8]Department of Microbiology and Immunology, School of Medicine, College of Medicine, Taipei Medical University, Taipei, Taiwan.

*Authors contributed equally to this work

[§]Corresponding author

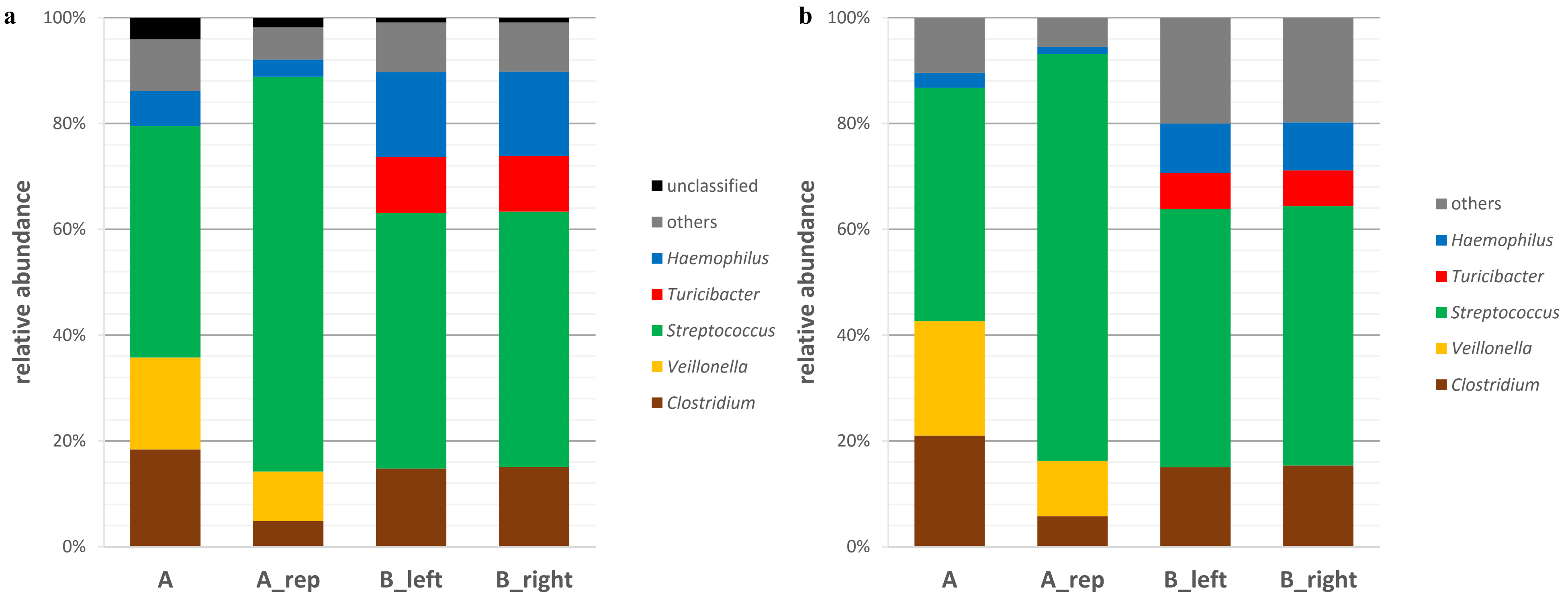

Supplementary Figure 1. Phylogenetic profiling for the four transcriptomes provided in SRP020487 (Leimena et al., 2013) from (a)FunctionAnnotator and (b)MG-RAST. Contigs assembled with CLC Genomics Workbench were upload to FunctionAnnotator and MG-RAST. The annotation results from MG-RAST were processed by SAMSA. Both the annotation results showed that the top five most dominate genus, *Streptococcus*, *Veillonella*, *Clostridium*, *Haemophilus*, and *Turicibacter* identified are the same as those reported in Leimena et al., 2013.

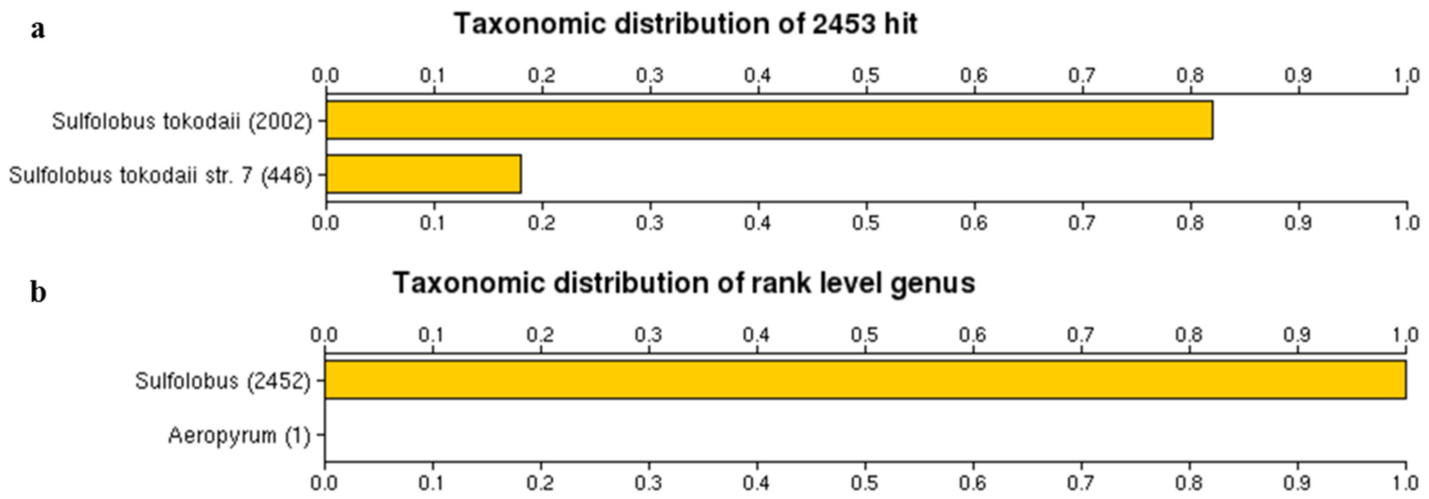

Supplementary Figure 2. Taxonomy distribution for the simulated transcriptome (*Sulfolobus tokodaii*) at (a) species level and (b) genus level. We uploaded 2,455 contigs to FunctionAnnotator and 2,452 of them have best hit in *Sulfolobus tokodaii* or organisms in the *Sulfolobus* genus.

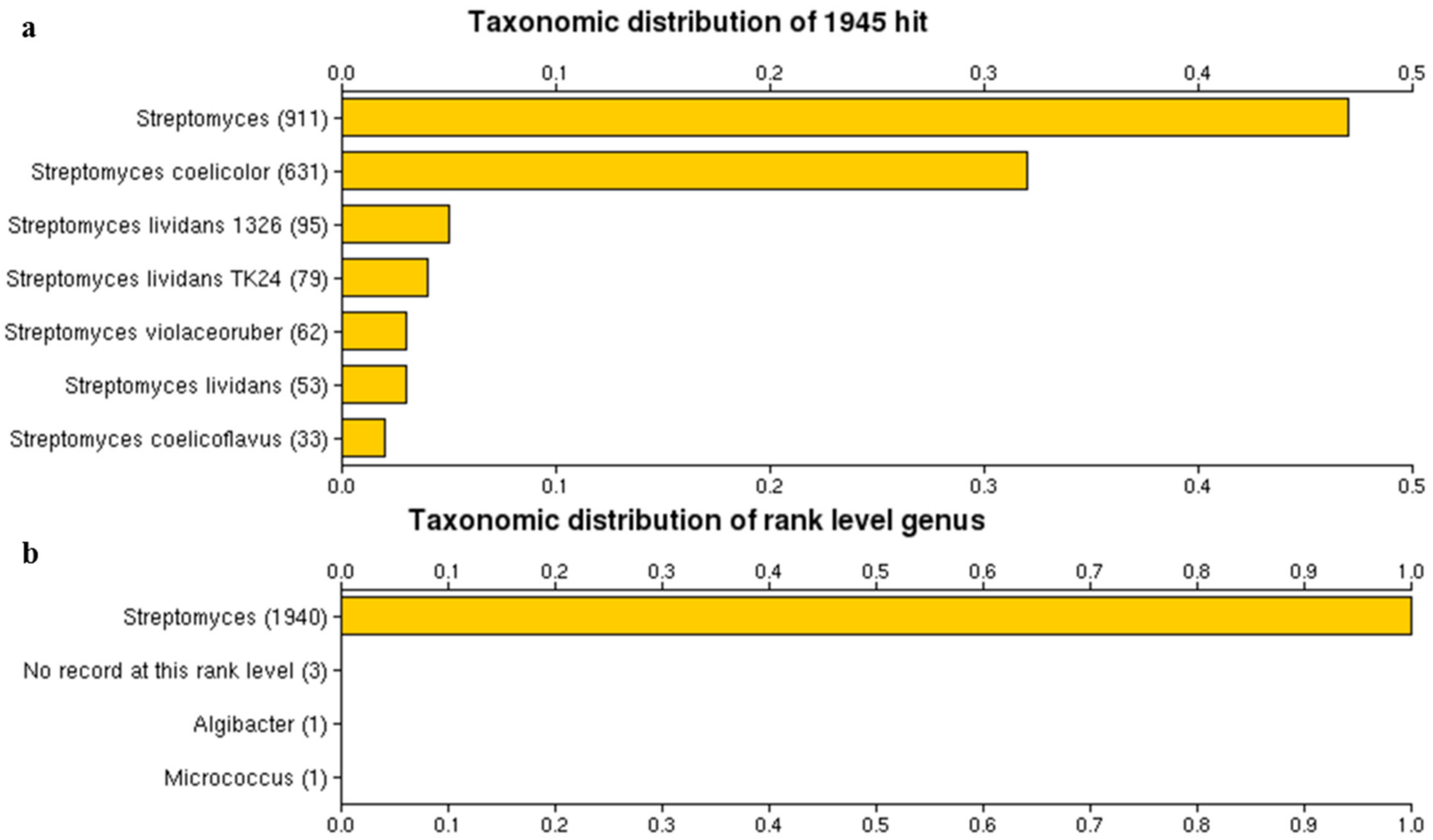

Supplementary Figure 3. Taxonomy distribution for the simulated transcriptome (*Streptomyces coelicolor*) at (a) species level and (b) genus level. We uploaded 1,945 contigs to FunctionAnnotator and all of them have best hit in *Streptomyces coelicolor* or organisms closely related to *Streptomyces coelicolor*.

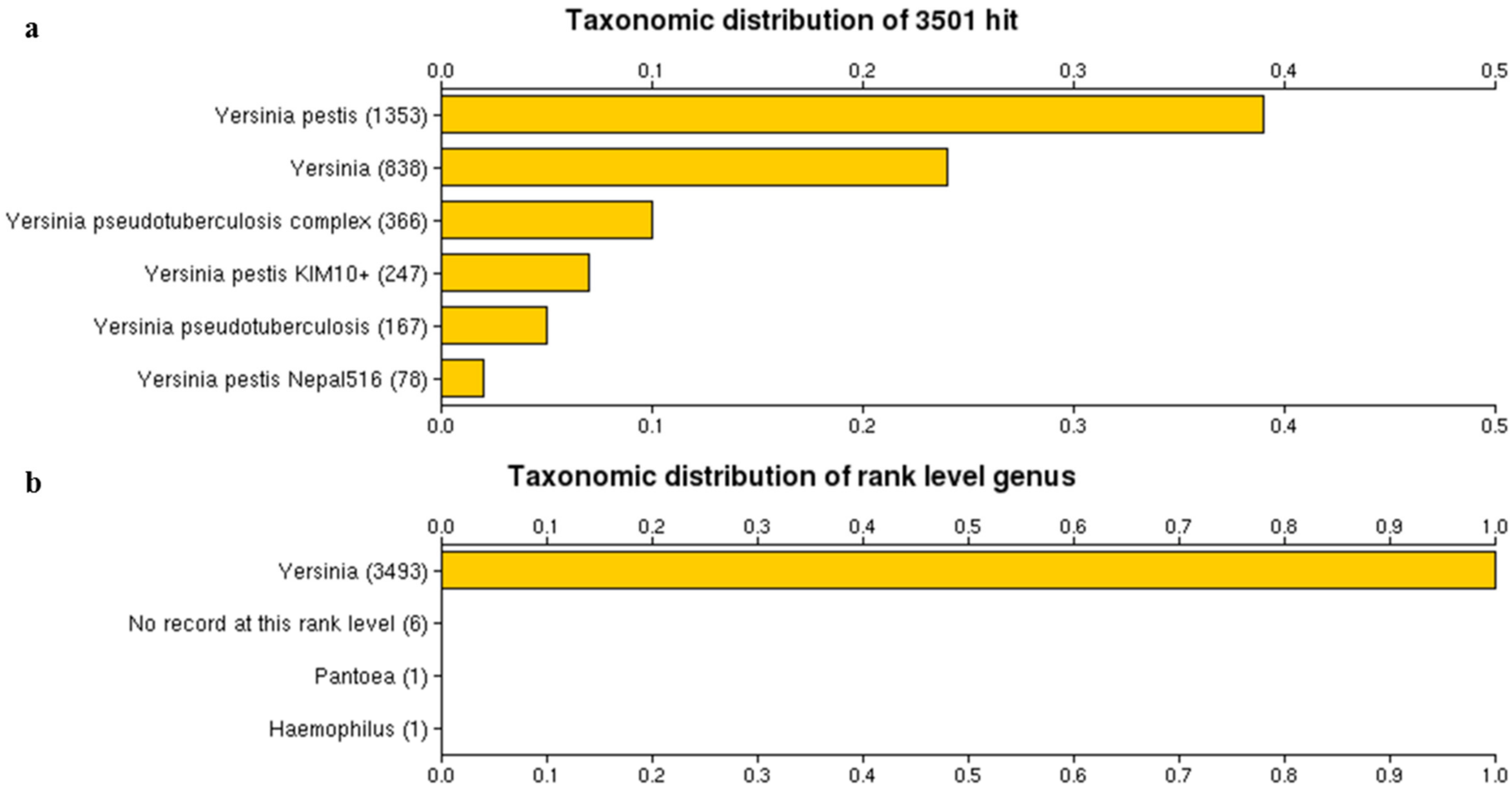

Supplementary Figure 4. Taxonomy distribution for the simulated transcriptome (*Yersinia pestis*) at (a) species level and (b) genus level. We uploaded 3,501 contigs to FunctionAnnotator and 3,493 of them have best hit in *Yersinia pestis* or organisms in the *Yersinia* genus.

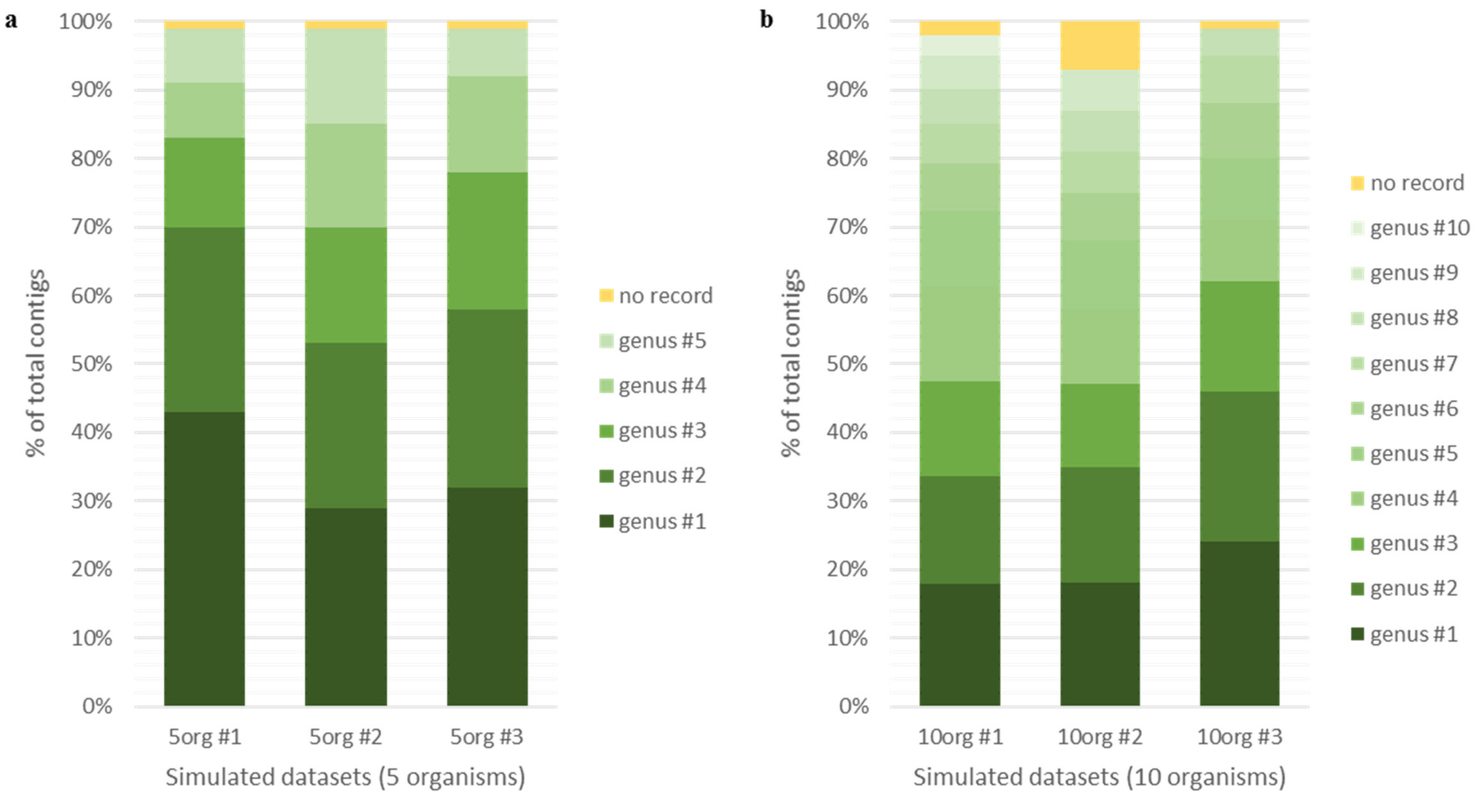

Supplementary Figure 5. FunctionAnnotator assigned genus taxonomy information for assembled contigs and identified all the genuses in our simulated metatranscriptome from (a) 5 organisms or (b) 10 organisms from FunctionAnnotator. The y-axis shows how the percentage of contigs that was assigned to the genus group. Green color represents correct assignment and yellow color represents no record at the genus level. Organisms belonging to the same genus were grouped together and shown with the same color. There are 5, 5, 5, 10, 9, 8 genus groups for 5org #1, 5org #2, 5org #3, 10org #1, 10org #2 and 10org #3, respectively.

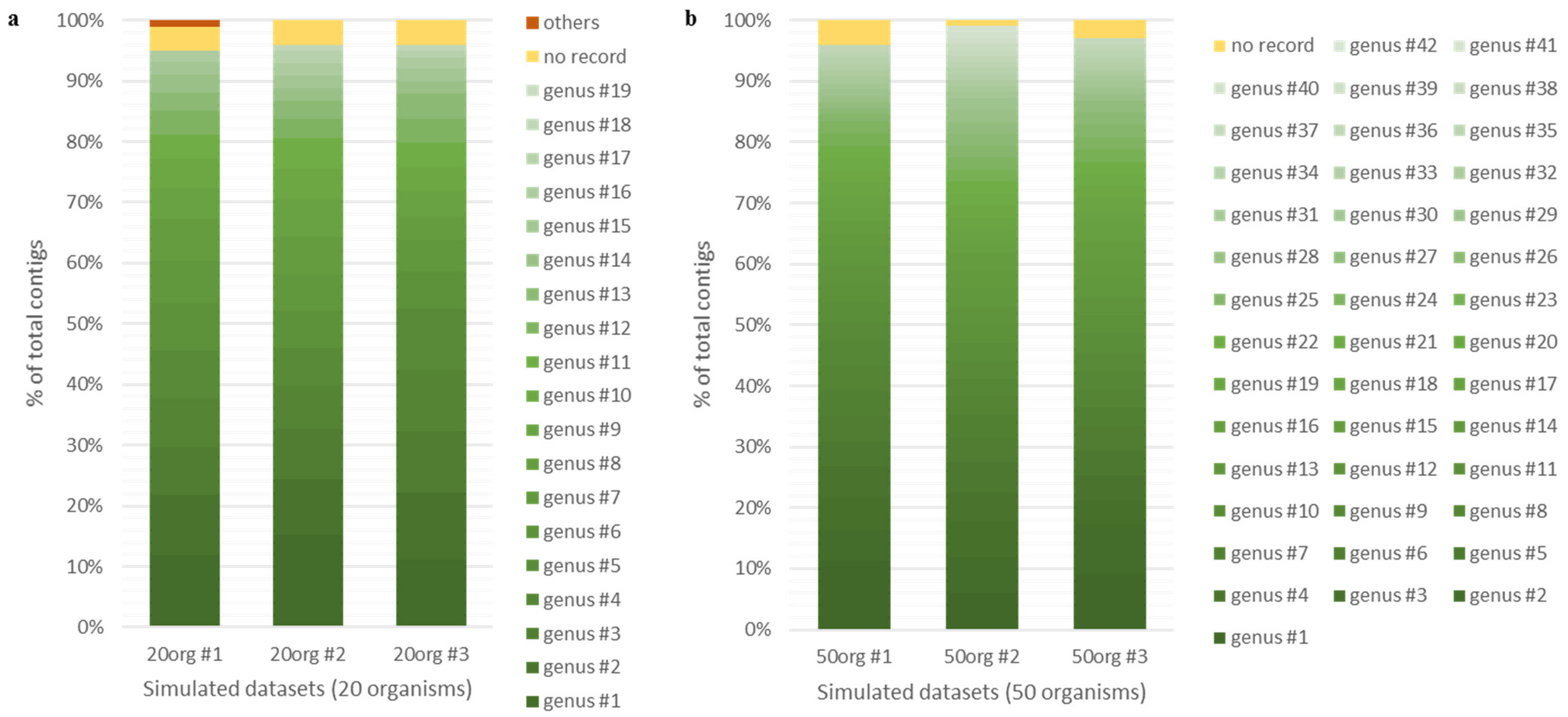

Supplementary Figure 6. FunctionAnnotator assigned genus taxonomy information for assembled contigs and identified all the genuses in our simulated metatranscriptome from (a) 20 organisms or (b) 50 organisms from FunctionAnnotator. The y-axis shows how the percentage of contigs were assigned to the genus group. Green color represents correct assignment, yellow color represents no record at the genus level and the brown color represents genus not in the simulated dataset. Organisms belonging to the same genus were grouped together and shown with the same color. There are 16, 18, 19, 37, 42 and 40 genus groups for 20org #1, 20org #2, 20org #3, 50org #1, 50org #2 and 50org #3, respectively.

Supplementary Table 1. Reference bacteria used in metatranscriptome simulation (I).

Dataset ID	Randomly selected bacteria
**5org #1**	*Candidatus Mycoplasma* haemolamae Purdue uid171259
	Geobacillus thermoleovorans CCB US3 UF5 uid82949
	Anaplasma phagocytophilum HZ uid57951
	Hydrogenobaculum HO uid190882
	Mycobacterium abscessus bolletii 50594 uid205422
**5org #2**	*Streptococcus suis* SS12 uid162123
	Oligotropha carboxidovorans OM5 uid59155
	Brucella abortus A13334 uid83615
	Thermofilum pendens Hrk 5 uid58563
	Arcobacter butzleri 7h1h uid200766
**5org #3**	*Amycolatopsis orientalis* HCCB10007 uid203791
	Mesorhizobium loti MAFF303099 uid57601
	Desulfovibrio africanus Walvis Bay uid66847
	Pseudomonas denitrificans ATCC 13867 uid195459
	Staphylococcus aureus 6850 uid217772

Supplementary Table 2. Reference bacteria used in metatranscriptome simulation (II).

Dataset ID	Randomly selected bacteria
**10org #1**	*Gordonia* sp. KTR9 uid174812
	Zymomonas mobilis pomaceae ATCC 29192 uid68445
	Zobellia galactanivorans uid70621
	Streptococcus pneumoniae D39 uid58581
	Chelativorans sp. BNC1 uid58069
	Propionibacterium acnes TypeIA2 P acn31 uid80733
	Staphylococcus aureus MW2 uid57903
	Spiroplasma apis B31 uid230613
	Pseudomonas fluorescens Pf0 1 uid57591
	Phaeobacter gallaeciensis uid54715
**10org #2**	*Streptococcus pneumoniae* INV200 uid162035
	Octadecabacter arcticus 238 uid54699
	Chlamydia trachomatis uid196778
	Prevotella denticola F0289 uid65091
	Maribacter sp. HTCC2170 uid51877
	Calothrix sp. PCC 7507 uid182930
	Chlamydia psittaci 01DC12 uid179070
	Escherichia coli O103 H2 12009 uid41013
	Staphylococcus aureus JKD6008 uid159855
	Bordetella bronchiseptica 253 uid178913
**10org #3**	*Mycoplasma gallisepticum* CA06 2006 052 5 2P uid172630
	Serratia sp. AS12 uid67315
	Thermotoga maritima MSB8 uid202924
	Desulfovibrio hydrothermalis AM13 DSM 14728 uid184831
	Mycoplasma gallisepticum R low uid57993
	Anabaena sp. 90 uid179383
	Bartonella vinsonii berkhoffii Winnie uid189951
	Mycoplasma gallisepticum NC06 2006 080 5 2P uid172629
	Haemophilus influenzae PittEE uid58591
	Thermofilum sp. 1910b uid215374

Supplementary Table 3. Reference bacteria used in metatranscriptome simulation (III).

Dataset ID	Randomly selected bacteria
20org #1	*Acinetobacter baumannii* ATCC 17978 uid58731
	Flavobacterium johnsoniae UW101 uid58493
	Chlamydia trachomatis E C599 uid222812
	Beijerinckia indica ATCC 9039 uid59057
	Ruminococcus sp. uid197156
	Thermosphaera aggregans DSM 11486 uid48993
	Salmonella bongori Sbon 167 uid213088
	Helicobacter pylori P12 uid59327
	Laribacter hongkongensis HLHK9 uid59265
	Escherichia coli APEC O78 uid187277
	Zunongwangia profunda SM A87 uid48073
	Escherichia coli O111 H 11128 uid41023
	Helicobacter pylori F16 uid161145
	Ehrlichia ruminantium Welgevonden uid58013
	Mesorhizobium ciceri biovar biserrulae WSM1271 uid62101
	Dehalococcoides mccartyi GY50 uid230266
	Stenotrophomonas maltophilia K279a uid61647
	Chlamydia pecorum P787 uid221292
	Sulfolobus islandicus Y N 15 51 uid58825
	Acinetobacter baumannii BJAB0868 uid210973
20org #2	*Bifidobacterium animalis* lactis Bi 07 uid163693
	Corynebacterium glutamicum MB001 uid214793
	Desulfotomaculum acetoxidans DSM 771 uid59109
	Amycolatopsis mediterranei S699 uid158689
	Clostridium phytofermentans ISDg uid58519
	Corynebacterium efficiens YS 314 uid62905
	Borrelia duttonii Ly uid58791
	Synechococcus sp. CC9605 uid58319
	Erysipelothrix rhusiopathiae SY1027 uid206518
	Salmonella enterica serovar Thompson RM6836 uid222802
	Acinetobacter baumannii ATCC 17978 uid58731
	Anaplasma marginale Maries uid57629
	Rickettsia conorii Malish 7 uid57633
	Klebsiella pneumoniae JM45 uid215235
	Mycobacterium tuberculosis CTRI 2 uid161997

	Thauera sp. MZ1T uid58987
	Citrobacter koseri ATCC BAA 895 uid58143
	Yersinia pestis Nepal516 uid58609
	Leuconostoc kimchii IMSNU 11154 uid48589
	Synechococcus elongatus PCC 7942 uid58045
	Bacillus cereus B4264 uid58757
	Campylobacter jejuni 81 176 uid58503
	Streptococcus anginosus C1051 uid218003
	Rhodospirillum rubrum ATCC 11170 uid57655
	Alpha-proteobacterium HIMB59 uid175778
	Ehrlichia canis Jake uid58071
	Nitrobacter hamburgensis X14 uid58293
	Alkaliphilus metalliredigens QYMF uid58171
	Sulfolobus islandicus M 14 25 uid58849
**20org #3**	*Bifidobacterium bifidum* S17 uid59545
	Candidatus Portiera aleyrodidarum BT QVLC uid175570
	Rickettsia canadensis McKiel uid58159
	Mycoplasma hyorhinis MCLD uid162087
	Neisseria meningitidis WUE 2594 uid162093
	Candidatus Nasuia deltocephalinicola NAS ALF uid214084
	Lactococcus lactis cremoris A76 uid160937
	Treponema pallidum Chicago uid159543
	Polymorphum gilvum SL003B 26A1 uid65447
	Porphyromonas gingivalis TDC60 uid67407
	Olsenella uli DSM 7084 uid51367

Supplementary Table 4. Reference bacteria used in metatranscriptome simulation (IV).

Dataset ID	Randomly selected bacteria
50org #1	*Helicobacter pylori* uid159983<br>*Rickettsia africae* ESF 5 uid58799<br>*Lactobacillus delbrueckii* subsp. *bulgaricus* ATCC BAA 365 uid57987<br>*Yersinia pestis* Nepal516 uid58609<br>*Enterobacter* sp. R4-368 uid208672<br>*Legionella pneumophila* Lens uid58209<br>*Sulfurospirillum deleyianum* DSM 6946 uid41861<br>*Chlamydia trachomatis* IU824 uid193712<br>*Rickettsia bellii* OSU 85 389 uid58681<br>*Salmonella bongori* NCTC 12419 uid70155<br>*Bacillus cereus* AH187 uid58753<br>*Pelagibacterium halotolerans* B2 uid74393<br>*Helicobacter pylori* Shi169 uid162209<br>*Acinetobacter oleivorans* DR1 uid50119<br>*Burkholderia mallei* ATCC 23344 uid57725<br>*Burkholderia* sp. RPE64 uid205541<br>*Lactococcus garvieae* Lg2 uid161935<br>*Mycobacterium tuberculosis* Haarlem uid54453<br>*Kitasatospora setae* KM 6054 uid77027<br>*Ignavibacterium album* JCM 16511 uid162097<br>*Francisella* cf *novicida* Fx1 uid162105<br>*Clostridium difficile* R20291 uid40921<br>*Pseudonocardia dioxanivorans* CB1190 uid65087<br>*Burkholderia phymatum* STM815 uid58699<br>*Streptococcus pyogenes* NZ131 uid59035<br>*Escherichia coli* W uid162101<br>*Chlamydia psittaci* MN uid175573<br>*Thermococcus gammatolerans* EJ3 uid59389<br>*Frankia* sp. EAN1pec uid58367<br>*Cycloclasticus* sp. P1 uid176368<br>gamma proteobacterium HdN1 uid51635<br>*Gordonia polyisoprenivorans* VH2 uid86651<br>*Neisseria meningitidis* WUE 2594 uid162093<br>*Streptococcus intermedius* JTH08 uid168614

	Salmonella enterica serovar Thompson RM6836 uid222802
	Mycoplasma synoviae 53 uid58061
	Rickettsia rickettsii Arizona uid86655
	Pseudomonas fluorescens F113 uid87037
	Bacillus cytotoxicus NVH 391 98 uid58317
	Slackia heliotrinireducens DSM 20476 uid59051
	Shewanella piezotolerans WP3 uid58745
	Methanocaldococcus jannaschii DSM 2661 uid57713
	Caldivirga maquilingensis IC 167 uid58711
	Verrucosispora maris AB 18 032 uid66297
	Natronobacterium gregoryi SP2 uid74439
	Thermotoga sp. RQ2 uid58935
	Streptococcus agalactiae NEM316 uid61585
	Salmonella enterica serovar Typhimurium U288 uid198746
	Coxiella burnetii RSA 331 uid58637
	Streptococcus parasanguinis FW213 uid163997
**50org #2**	*Helicobacter cetorum* MIT 99 5656 uid162215
	Synechococcus sp. PCC 7002 uid59137
	Bacillus cereus biovar anthracis CI uid50615
	Synechococcus sp. PCC 7502 uid183008
	Shewanella pealeana ATCC 700345 uid58705
	Salmonella enterica serovar Bovismorbificans 3114 uid218006
	Corynebacterium diphtheriae CDCE 8392 uid84295
	Actinosynnema mirum DSM 43827 uid58951
	Borrelia burgdorferi ZS7 uid59429
	Streptococcus pneumoniae SPN034183 uid197186
	Gardnerella vaginalis 409 05 uid43211
	Alicyclobacillus acidocaldarius DSM 446 uid59199
	Baumannia cicadellinicola Hc (*Homalodisca coagulate*) uid58111
	Exiguobacterium antarcticum B7 uid176125
	Mannheimia haemolytica M42548 uid198769
	Campylobacter coli 15 537360 uid226113
	Pedobacter heparinus DSM 2366 uid59111
	Pasteurella multocida HN06 uid156881
	Pseudomonas monteilii SB3078 uid232252
	Helicobacter pylori Shi112 uid162207
	Spiroplasma syrphidicola EA 1 uid205054

	Propionibacterium acnes TypeIA2 P acn17 uid80735
	Bifidobacterium breve UCC2003 uid193702
	Serratia symbiotica Cinara cedri uid82363
	Roseobacter litoralis Och 149 uid54719
	Vibrio furnissii NCTC 11218 uid82347
	Riemerella anatipestifer RA GD uid162013
	Helicobacter pylori uid159983
	Staphylococcus aureus MSHR1132 uid89393
	Vibrio cholerae O395 uid58425
	Helicobacter pylori UM298 uid213226
	Methanothermobacter thermautotrophicus Delta H uid57877
	Cyanobium gracile PCC 6307 uid182931
	Magnetococcus sp. MC-1 uid57833
	Amycolatopsis orientalis HCCB10007 uid203791
	Caldicellulosiruptor bescii DSM 6725 uid59201
	Streptococcus pseudopneumoniae IS7493 uid71153
	Prevotella melaninogenica ATCC 25845 uid51377
	Mycoplasma gallisepticum NC06 2006 080 5 2P uid172629
	Aeromonas salmonicida A449 uid58631
	Burkholderia sp. CCGE1002 uid42523
	Candidatus Blochmannia pennsylvanicus BPEN uid58329
	Rhodospirillum photometricum uid159003
	Prochlorococcus marinus AS9601 uid58307
	Aggregatibacter actinomycetemcomitans D11S 1 uid41333
	Acidovorax citrulli AAC00-1 uid58429
	Yersinia pestis biovar Medievalis Harbin 35 uid158537
	Bacillus licheniformis 9945A uid207072
	Methanosarcina barkeri Fusaro uid57715
	Chlorobium phaeovibrioides DSM 265 uid58129
	Croceibacter atlanticus HTCC2559 uid49661
	Enterococcus faecalis OG1RF uid54927
	Helicobacter pylori Aklavik86 uid182202
**50org #3**	*Gloeobacter violaceus* PCC 7421 uid58011
	Bacteroides uniformis uid13130
	Lactococcus lactis KLDS 4 0325 uid225028
	alpha proteobacterium HIMB59 uid175778
	Bacillus cytotoxicus NVH 391 98 uid58317

Azospirillum brasilense Sp245 uid162161
Lactobacillus salivarius CECT 5713 uid162005
Bifidobacterium longum infantis ATCC 15697 uid159865
Pleurocapsa sp. PCC 7327 uid183006
Dehalogenimonas lykanthroporepellens BL DC 9 uid48131
Francisella tularensis TIGB03 uid89379
Salmonella enterica serovar Choleraesuis SC B67 uid58017
Phaeobacter gallaeciensis DSM 17395 uid54717
Acetobacter pasteurianus IFO 3283 26 uid158531
Lactobacillus acidophilus 30SC uid63605
Bacillus subtilis RO NN 1 uid158879
Listeria monocytogenes SLCC2479 uid175108
Vibrio fischeri MJ11 uid58907
Mycobacterium bovis BCG Mexico uid86889
Geobacter metallireducens GS 15 uid57731
Methylobacillus flagellatus KT uid58049
Blattabacterium Blatta orientalis Tarazona uid188115
Salmonella enterica serovar Enteritidis P125109 uid59247
Thalassobaculum sp. L2 uid182483
Modestobacter marinus uid167487
Burkholderia pseudomallei 1710b uid58391
Veillonella parvula DSM 2008 uid41927
Corynebacterium diphtheriae CDCE 8392 uid84295
Corynebacterium glutamicum R uid58897
Desulfohalobium retbaense DSM 5692 uid59183
Prochlorococcus marinus CCMP1375 uid57995
Helicobacter pylori Shi417 uid162205
Escherichia coli W uid162101
Desulfarculus baarsii DSM 2075 uid51371
Caulobacter crescentus NA1000 uid59307
Helicobacter pylori Shi112 uid162207
Dehalococcoides mccartyi DCMB5 uid190184
Treponema pallidum SS14 uid58977
Azospirillum lipoferum 4B uid82343
candidate division SR1 bacterium RAAC1 SR1 1 uid230714
Bacillus thuringiensis serovar kurstaki HD73 uid189188
Borrelia burgdorferi N40 uid161241

	Vibrio anguillarum 775 uid68057
	Mycoplasma genitalium M6282 uid173371
	Sphaerobacter thermophilus DSM 20745 uid41997
	Gluconobacter oxydans H24 uid179202
	Chlamydia trachomatis RC L2 s 46 uid213386
